# Rupture and hemorrhage of a seminoma mixed with yolk sac tumors in 46XY partial gonadal dysgenesis: a case report and literature review

**DOI:** 10.1186/s12893-021-01302-3

**Published:** 2021-07-03

**Authors:** Rui Lin, Nanbin Liu, Xiuyan Wang, Xuyou Zhu, Daojing Huang, Baomin Shi

**Affiliations:** 1 General Surgery Department, Tongji Hospital, Tongji University School of Medicine, Shanghai, 200065 China; 2 Ultrasound Department, Tongji Hospital, Tongji University School of Medicine, Shanghai, 200065 China; 3 Pathology Department, Tongji Hospital, Tongji University School of Medicine, Shanghai, 200065 China

**Keywords:** 46XY DSD, Partial gonad dysgenesis, Ambiguous genitalia, Diagnosis, Case report

## Abstract

**Background:**

46XY partial gonadal dysgenesis (PGD) is a rare subtype of disorder of sex development (DSD). 46YY PGD is a congenital disease with atypical chromosomal, gonadal, or anatomical sex development. The patient in this case report had male and female genitalia simultaneously. We created a flowchart of the differential diagnosis for clinicians.

**Case presentation:**

A 41-year-old male was admitted to the hospital complaining of lower quadrant abdominal pain for 1 day. Physical examination revealed that his penis size was normal, but a urethral orifice was located in the perineum area between the scrotum and anus. One small testicle was in the left scrotum, but no testicle was present on the right. The patient’s abdomen was bulging, and he had lower abdominal pain. According to the emergency CT scan, a lesion (74*65 mm) was found in the right pelvis between the bladder and rectum. The lesion showed an unclear boundary and hematocele appearance. The lesion was removed by emergency surgery, and the pathology report indicated a mixed germ cell tumor with a seminoma and yolk sac tumors.

**Conclusion:**

This article is a case report of germ cell tumors in 46XY PGD patients. The literature review summarizes the clinical diagnosis, and a flowchart is provided for physicians in future practice. The importance of this report is that it will help acquaint physicians with this rare disease and make the right initial clinical decision quickly through the use of this flowchart. However, the variants of special subtypes of 46XY DSD are myriad, and all the diagnoses could not be covered in one flowchart.

## Background

Sex development is a highly precise process and is regulated by many factors. Genetic factors, hormone levels and changes in the external environment may cause disorders of sex development (DSDs) [1]. DSDs can be classified as (1) sex chromosome-related, (2) 46XX-related, or (3) 46XY-related [1]. DSD is a disease of gonadal dysfunction caused by the interruption of gonadal development. The disease encompasses various phenotypes. The 46XY DSD phenotypes normally include ambiguous genitalia, streak gonads, varying degrees of hypospadias, cryptorchidism, abnormal development of pubic hair and gynecomastia [2]. According to the hormone level, 46XY DSD is subdivided into complete gonadal dysgenesis (CGD), partial gonadal dysgenesis (PGD), congenital adrenal hyperplasia (CAH), and androgen insensitivity syndrome (AIS) [3]. Some studies illustrate that the presence of the Y chromosome in 46XY DSD patients can increase the risk of developing gonadal tumors up to 50% [4]. Individuals who were diagnosed with 46XY DSD showed overlapping biochemical and clinical parameters. This requires further evaluation by multidisciplinary coordination. There is no consensus in the management of gonadal tumors for 46,XY DSD patients. To prevent the development of malignancy in these patients, orchidopexy at an early age is typically recommended.

The case in this article is rare. The patient was diagnosed with 46XY PGD with the rupture of a seminoma mixed with yolk sac tumors and had male and female genitalia simultaneously. After informing the patient and acquiring his consent, we deidentified the personal information and placed the unique radiology images and the patient’s photos in this article. We also reviewed the literature and summarized a diagnostic flowchart for clinicians.

## Case presentation

**Fig. 1:**
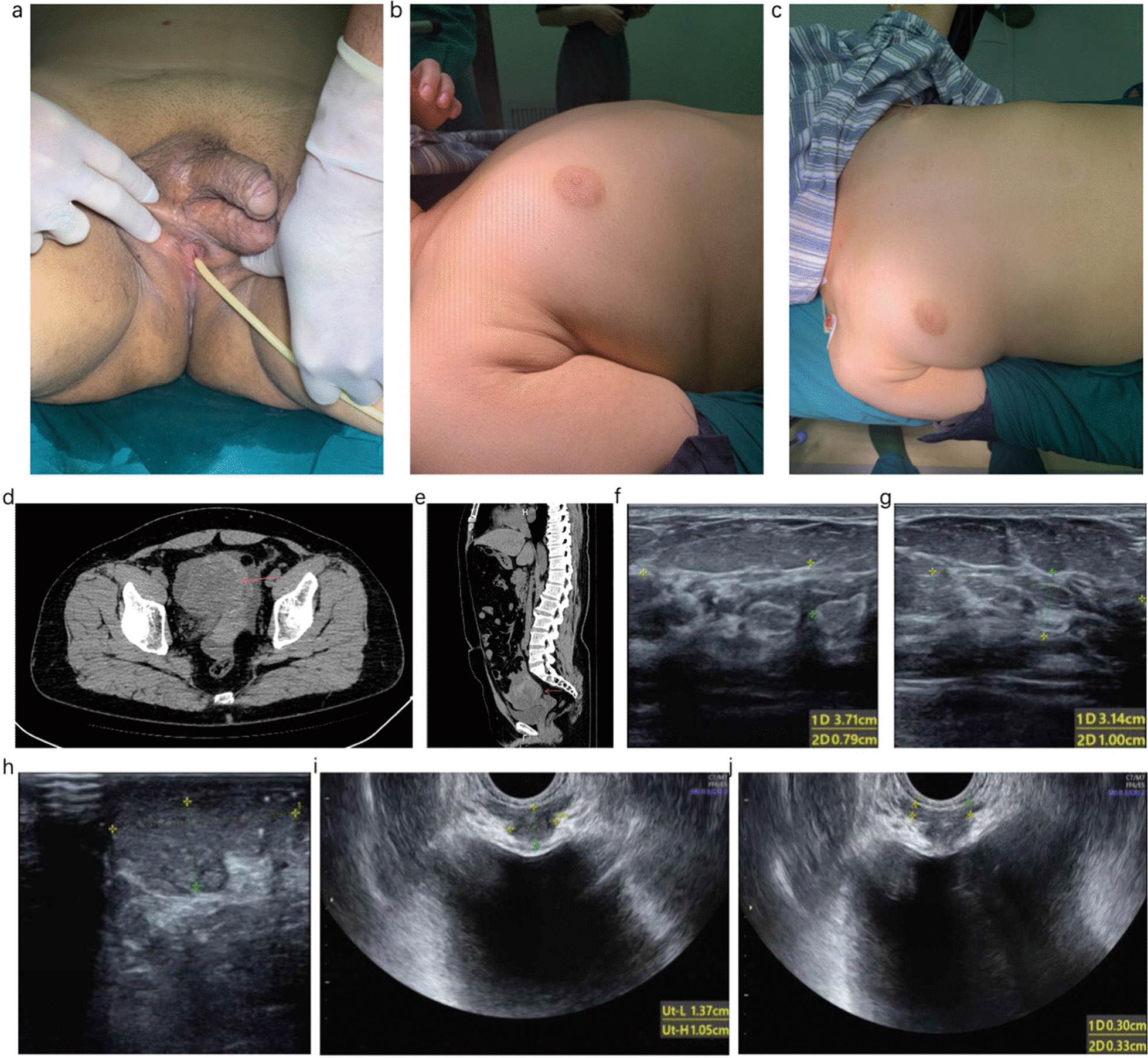
**a** Ambiguity of external genitalia with normal size penis and female-like urethral opening in the perineum area. **b** and **c** Gynecomastia (Grade II, Rohirich Classification) [5]. **d** The transverse plane of CT of the pelvis revealed a tumor located in the right pelvis (red arrow). **e** The sagittal plane of CT revealed a tumor (red arrow) located between the bladder and rectum. **f** and **g** Ultrasound of bilateral mammary gland. **h** Ultrasound of the left testicle. **i** Ultrasound of prostate-like structure in patient’s prostate area. **j** Ultrasound of bilateral seminal vesicle gland

A 41-year-old male patient was admitted to the emergency room on the afternoon of September 30th, 2020 due to the complaint of “lower abdominal pain for 1 day, aggravating for 2 h”. He had mild nausea and vomiting without diarrhea, bloody stool/urine or fever. He reported no familial tumor history or hereditary disease, except ambiguity external genitalia with a normal size penis and a female-like urethral orifice located in the perineum between the anus and scrotum (Fig. 1a). He also reported bilateral cryptorchidism and a surgical history of left orchiopexy at 9 years old. He had a normal libido, normal erectile function (International Index of Erectile Function score: 21 points), and good morning erections. There was no vaginal orifice or clitoris in his perineum. He also reported no menstrual cycle. His job had no exposure to cytotoxic agents or radiation, and he took no special medications.

Physical examination revealed that the height and weight of the patient were 167 cm and 70 kg, respectively. His blood pressure was 116/70 mmHg. He presented sparse body hair and bilateral gynecomastia (Grade II, Fig. 1b, c). The lower quadrant of the abdomen was tender without rebounding pain. Bowel sounds were normal, with a frequency of 8 per minute. Genital examination showed symmetrical male external genitalia with a stretched penis length of 7 cm. There was no urethral opening on the tip of penis. Otherwise, the urethral orifice was located in the perineum between the scrotum and anus. The left testicle could be touched in the left-sided scrotum, with a small volume of 9 mL. The right testicle was absent in the right-sided scrotum. Pubic hair was sparse (Tanner Stage II, Fig. 1a). Digital rectal examination revealed a small prostate gland (approximately 2 cm) and fluctuation at the tip of the finger.

An abdominal CT scan showed a solid tumor located in the right pelvis between the bladder and rectum with a hematocele and unclear boundary (Fig. 1d, e). Pelvic ultrasound revealed a solid tumor located on the posterior part of the bladder with a size of 74*65 mm. There was a prostate-like echo in the prostate region with a range of 14*11 mm (Fig. 1i). The echo of the bilateral seminal vesicle gland was visible, and the anteroposterior size was 3 mm with decreased internal echogenicity (Fig. 1j). The left testicle was small (24*11 mm), with microlithiasis (Fig. 1h), and the right scrotum was empty. Mammary zone ultrasound showed fibroglandular-like echogenicity with a right-side range of 37*8 mm and a left-side range of 32*10 mm (Fig. 1f, g).

Laboratory test results were as follows: hemoglobin (Hb), 80 g/L(130–175 g/L); red blood cell (RBC) count, 4.25 × 10^12^/L (4.3–5.8 × 10^12^/L); platelet (PLT) count, 214 × 10^9^/L (125–350 × 10^9^/L); white blood cell (WBC) count, 13.68 × 10^9^/L (3.5–9.5 × 10^9^/L); alpha fetoprotein (AFP), > 1210 ng/mL (< 7 ng/mL); human chorionic gonadotropin (HCG), 898 mIU/mL (< 5 mIU/mL); and lactate dehydrogenase (LDH), 2583 U/L (313–618 U/L).

The results of hormone analysis were as follows: follicle-stimulating hormone (FSH), 20.8 mIU/mL (1–13 mIU/mL); luteinizing hormone (LH), 9.79 mIU/mL (1–8.4 mIU/mL); early morning total testosterone (T), 0.54 nmol/l (4.27–28.24 nmol/L); free testosterone (FT), 1.75 pg/mL (15–50 pg/mL); and serum dihydrotestosterone (DHT); 40 pg/mL (112–955 pg/mL). Estradiol (E2) and prolactin (PRL) levels were 0.8 nmol/L (normal range in males: < 19 nmol/L) and 15.32 (2.1–17.7 µg/L), respectively. The short-term hCG stimulating test, in which the patient was given 3000 IU/m^2^ intramuscular injection for 3 continuous days, showed that the 1st and 4th day testosterone levels after the test were 1.53 and 2.05 nmol/L, respectively.

A karyotype of 46,XY was found by testing peripheral blood lymphocytes. We proposed a testicular biopsy to the patient to obtain a histological analysis, but the proposal was refused by the patient.

**Fig. 2:**
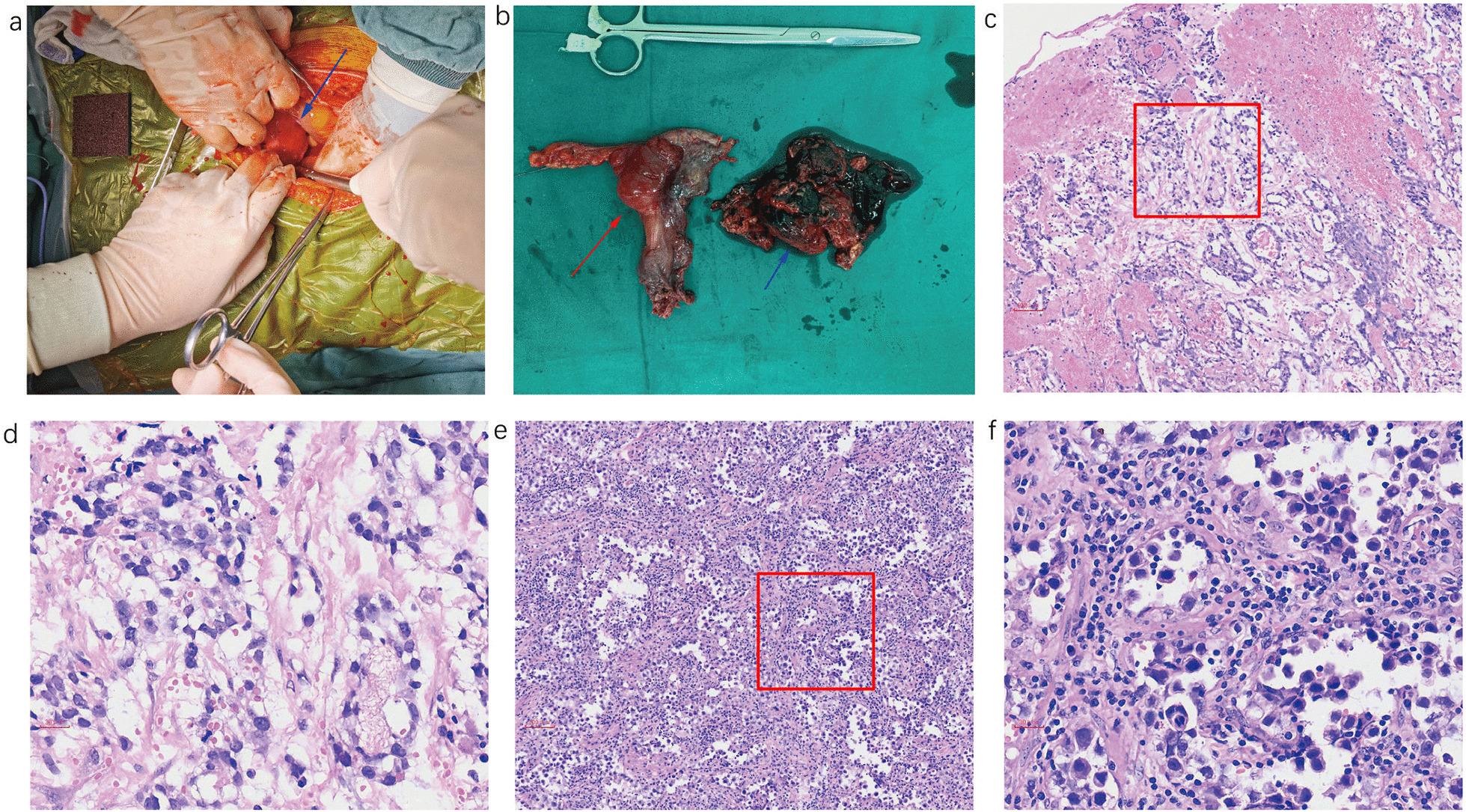
**a** Photograph of surgery with midline incision. The blue arrow indicates the tumor and uterus-like structure in the right pelvis. **b** Specimens resected by surgery. Uterus-like structure (red arrow) and ruptured tumor (blue arrow) Pathology of the tumor: **c** Yolk-sac tumors: Loose network of anastomosing channels that focally expand to form variably sized cysts (H&E, X100). **d** The high-power magnification image from **c** (red rectangular box) shows that the cysts of yolk sac tumors are lining with transparent or flat atypical epithelium (H&E, X400). **e** Seminoma: low-power magnification showing nests of primitive germ cells involved by sex cord cells and hyaline basal membrane material. **f** The high-power magnification image from **e** (red rectangular box) shows that the tumor cells are round or polygon shaped. The tumor cells had clear cytoplasm and large nuclei. The nuclear membrane and chromatin are bulky, and mitotic phenomena are rare on imaging (H&E, X400)

The patient was diagnosed with a disorder of sex development, acute lower abdominal pain and suspected pelvic tumor rupture. He underwent emergency surgery immediately after admission to the ward. We explored his abdominal area and found that the ruptured tumor was located on the right side of the pelvis, with a uterus-like structure between the bladder and the rectum. No ovary was found in the abdomen. Eight hundred milliliters of free uncoagulated blood was collected in the abdomen. The 8 by 10 cm uterus-like structure with the ruptured tumor (Fig. 2a, b) was resected with 400 mL blood infusion. After resection, the blood pressure and hemoglobin level were stabilized.

The pathological results showed that the tumor was a seminoma (mainly) mixed with nests of yolk sac tumors (Fig. 2c–f. The figures were taken by MoticEasyScan with its build-in software). Immunohistochemical results were as follows: CD117 (+), CEA (−), AFP (+), β-HCG (+), CKpan (partial +), Ki-67 (+ approximately 80%), P53 (+), PLAP (+), CD30(−), EMA (small amount +), S-100 (partial +), CD34 (blood vessel +), PD-L1 (+ 30%), C-erbB-2 (0+), MLH1 (+), PMS2 (+), MSH2 (+), and MSH6 (+).

## Discussion and conclusion

The inconsistency among genetic (XY or XX), gonadal (ovaries or testis), external genitalia (penis or vulva), and internal genitalia (Mullerian or Wolffian ducts) causes DSD [1]. 46XY DSD is a kind of testicular development disorder and androgen synthesis or function disorder. There are several subcategories of 46XY DSD, as follows [6].

1. Patients with complete or partial gonadal dysgenesis (CGD or PGD) have disorders of testicular development.
2. Congenital adrenal hyperplasia (CAH) is caused by mutations in the 17β-HSD3 or SRD5A2 genes and is involved in androgen synthesis disorders. The 17β-HSD3 gene mutation can cause deficiency in the conversion of androstenedione to testosterone. SRD5A2 gene mutation can cause deficiency in 5α-reductase that can make the conversion of testosterone to its more potent form of dihydrotestosterone (DHT).
3. Androgen receptor gene mutations can lead to androgen insensitivity syndrome (AIS), which also includes partial (PAIS) or complete (CAIS) forms.

The first-line testing in newborns for diagnosing 46XY DSD should include karyotyping, imaging (ultrasound/CT/MRI), and serum hormone analysis. Molecular testing for gene mutations is limited by accessibility and cost [1]. The differential diagnosis should be based on clinical presentation (Table 1), imaging findings, and hormone tests.

**Table 1:** Differential diagnosis of 46XY DSD

Clinical presentation	Disorder of testicular development		Androgen synthesis disorder		Androgen receptor mutation	
	CGD	PGD	17β-HSD3 mutation	SRD5A2 mutation	CAIS	PAIS
External genitalia	Female	Ambiguous	Female or ambiguous	Ambiguous	Female	Ambiguous
Gonads	Streak gonads	Small testicles	Small testicles	Small to normal testicles	Normal	Normal
Hypospadias	No	Yes	Yes	Yes	No	Yes
Body hair	Sparse	Varied	Sparse	Sparse	Sparse	Varied
Gynecomastia	Yes	Yes	Yes	Yes	Yes	Yes
Cryptorchidism	Yes	Yes	Yes	Yes	Yes	Yes

*CGD* complete gonad dysgenesis, *PGD* partial gonad dysgenesis, *CAIS* complete androgen insensitivity syndrome, *PAIS* partial androgen insensitivity syndrome

The clinical presentation of 46XY CGD includes unambiguous female genitalia, bilateral streak gonads, and amenorrhea [7]. PGD patients may have a wide spectrum of phenotypes associated with varying degrees of hypospadias, ambiguous genitalia, cryptorchidism, and variable testis sizes [8]. DSD patients with the 17β-HSD3 gene mutation could display complete or predominantly female genitalia, cryptorchidism, hypospadias, gynecomastia, and sparse body hair [9]. Most 17β-HSD3 gene mutation patients were female, but they had Wolffian derivatives such as an epididymis, vas deferens, seminal vesicles, and ejaculatory ducts. SRD5A2 gene mutation patients have ambiguous genitalia, variable testis size, hypospadias, sparse body hair, cryptorchidism, and gynecomastia [10]. CAIS patients have complete female genitalia with primary amenorrhea, normal breast development, sparse body hair, and cryptorchidism, but they have no uterus or ovaries [11]. PAIS patients have ambiguous genitalia ranging from complete male to complete female forms. PAIS always features micropenis, hypospadias, bifid scrotum, and cryptorchidism [12].

In clinical circumstances, ultrasound, CT or MRI are the most commonly used tools to determine the internal genitalia status of DSD patients. For CGD patients, there is usually no uterus or just a rudimentary uterus in the pelvis. Undescended streak gonads, Wolffian remnants, or fallopian tubes could also be found [7]. The imaging findings in PGD are similar to those of CGD but could range from normal male internal genitalia to a rudimentary uterus with a blind-ended vagina. The gonads could be dysgenesis testes located along the path from pelvic descending to scrotum [8]. Most of the 17β-HSD3 mutation patients have bilateral cryptorchidism and Wolffian derivatives, as with SRD5A2 gene mutation, CAIS, and PAIS patients. Mullerian derivatives are always absent from the imaging findings [9–12] in these subcategories.

**Fig. 3:**
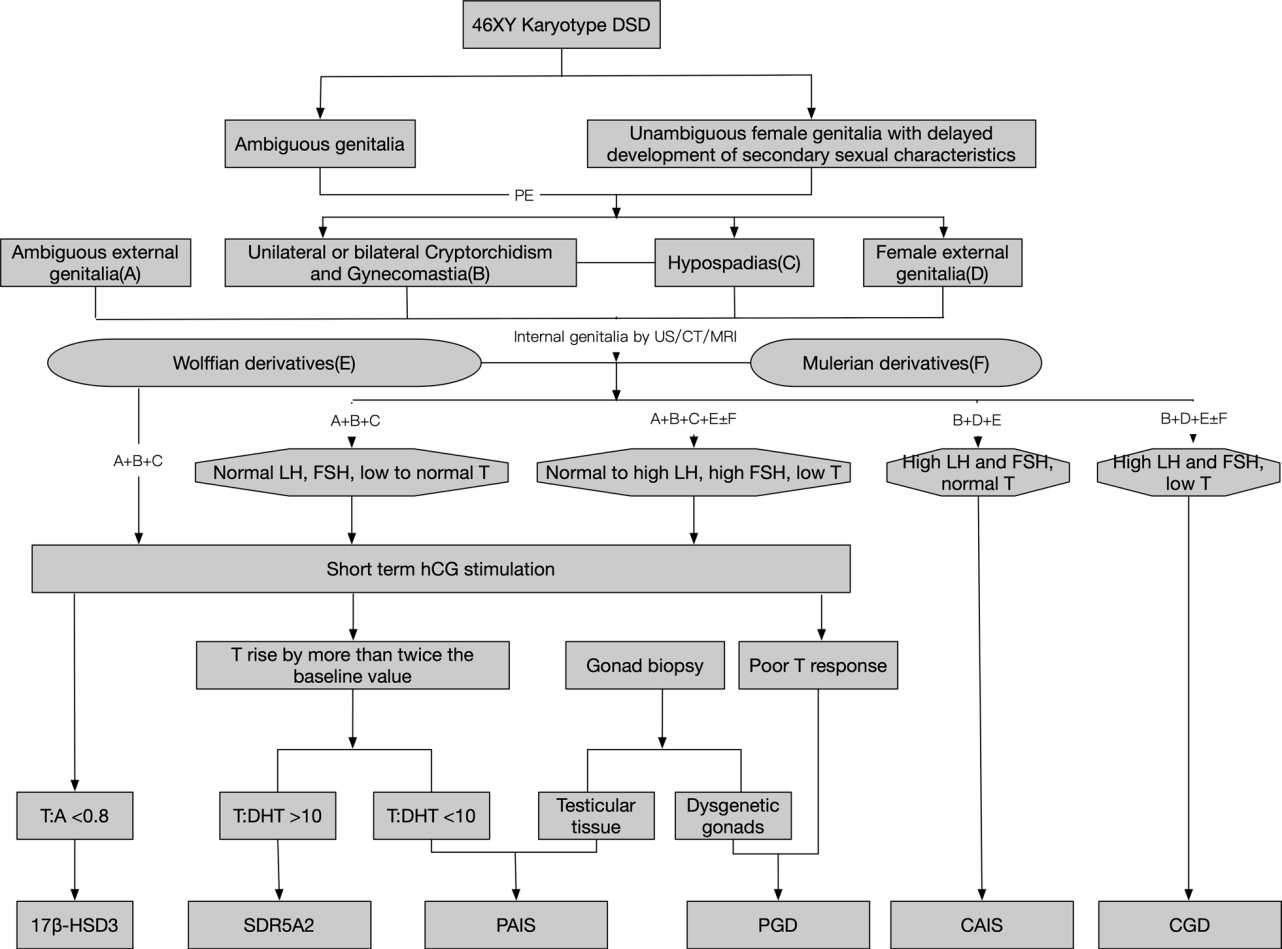
Flowchart for 46XY DSD differential diagnosis. *DSD* disorders of sex development, *PE* physical examination, *US* ultrasound, *CT* computed tomography, *MRI* magnetic resonance image, *LH* luteinizing hormone, *FSH* follicle-stimulating hormone, *T* testosterone, *hCG* human chorionic gonadotropin, *A* androstenedione, *DHT* dihydrotestosterone, *17βHSD3* 17β-hydroxysteroid dehydrogenase 3, *PAIS* partial androgen insensitivity syndrome, *PGD* partial gonad dysgenesis, *CAIS* complete androgen insensitivity syndrome, *CGD* complete gonad dysgenesis

The most important diagnostic tool for 46XY, DSD, is hormone testing. For CGD patients, gonadotropin hormones (LH and FSH) are elevated in the absence of gonadal steroid production [6]. In comparison, although PGD patients have elevated gonadotropin, the gonadal steroid level depends on the degree of testicular tissue present [8]. Short-term hCG administration to PGD patients shows a poor T response. The gonadal biopsy will reveal unilateral or bilateral dysgenesis gonads in PGD patients. For 17β-HSD3 mutation patients, the ratio of testosterone (T) to androstenedione (A) was less than 0.8 after short-term hCG stimulation. This is the diagnostic criterion for 17β-HSD3 mutation DSD [9]. For SRD5A2 gene mutation patients, there will be more than twice the value of the baseline of T elevation after short-term hCG stimulation [10]. The ratio of T to DHT (dihydrotestosterone) is always more than 10 in this kind of DSD patient when hCG stimulation is administered. This is the differential criterion between SRD5A2 gene mutation and PAIS DSD. In PAIS patients, the T to DHT ratio is always less than 10, and gonadal biopsy is a testicular issue [12]. However, PAIS patients share a similar biochemical feature with SRD5A2 gene mutation patients. PAIS patients are found to have normal LH and FSH and low to normal T. The hormone test criteria for CAIS are much easier than those for other kinds of DSD. The gonadotropins are elevated, and the testosterone is normal [11]. The flowchart for 46XY disorders of sexual development differential diagnosis presents the details for clinicians (Fig. 3).

In this report, the patient had ambiguous external genitalia, gynecomastia, right-sided cryptorchidism and sparse body hair. Wolffian derivatives (prostate and bilateral seminal vesicle) were revealed by pelvic ultrasound. A solid tumor was also detected on the posterior part of the bladder. The image of the tumor after resection shown in Fig. 2a, b indicated that it was a uterus-like structure with a ruptured tumor. The pathology results verified that it was a seminoma (mainly) mixed with nests of yolk sac tumors (Fig. 2c–f). We believe that the uterus-like structure was a Mullerian derivative, and the ruptured tumor was of right cryptorchidism origin. The hormone test revealed that the levels of FSH and LH were high and testosterone was low, with a poor response in the short-term hCG stimulating test. Based on the above evidence, the patient was diagnosed with 46XY partial gonad dysgenesis with a ruptured seminoma tumor mixed with yolk sac tumors.

46XY PGD may be hard to diagnose because the clinical presentation, laboratory test, and imaging findings are usually not consistent with the theory. There are some special subtypes of 46XY DSD that always confuse clinicians. For example, ovotesticular DSD (OT-DSD) patients are very similar to PGD patients in terms of clinical presentations, image findings, and biochemical profiles. The only difference is a rare condition with both ovarian and testicular tissue simultaneously present in an individual after histopathological examination [13]. There is also a rare subtype of congenital bilateral anorchia (CBA). It is also very similar to PGD patients except that there was no T response after short-term HCG stimulation [14]. These rare subtypes are not included in our diagnostic flowchart because they will further complicate the flowchart. The hormonal test and karyotype are the key for the differentiated diagnosis. Variable presentations and imaging findings are also important. The flowchart will be a useful tool for clinicians to make the right initial diagnosis quickly. However, we still need further differential diagnosis for the very rare types after the initial diagnosis is made.

In this report, we present the case of a 46,XY PGD patient with a ruptured germ cell tumor and review the literature to summarize the differential diagnostic flowchart for 46,XY DSD patients. This flowchart is easy to use and will help clinicians make the initial diagnosis. However, our diagnostic flowchart does not include all the rare subtypes of 46XY DSD.

## Data Availability

All datasets generated or analyzed during this study are available from the corresponding author on reasonable request.

## Abbreviations

DSD: Disorder of sex development
PE: Physical examination
US: Ultrasound
CT: Computed tomography
MRI: Magnetic resonance image
LH: Luteinizing hormone
FSH: Follicle-stimulating hormone
T: Testosterone
hCG: Human chorionic gonadotropin
A: Androstenedione
DHT: Dihydrotestosterone
17βHSD3: 17β-Hydroxysteroid dehydrogenase 3
PAIS: Partial androgen insensitivity syndrome
PGD: Partial gonad dysgenesis
CAIS: Complete androgen insensitivity syndrome
CGD: Complete gonad dysgenesis
